# Posterior reversible encephalopathy syndrome associated with antibiotic therapy: a case report and systematic review

**DOI:** 10.1007/s10072-024-07545-1

**Published:** 2024-04-29

**Authors:** Lorenzo Barba, Carmelo Carrubba, Kai Spindler, Christopher M. Weise, Torben Sachs, Matteo Foschi, Lucio D’Anna, Bernhard Sehm, Richard Ibe, Erck Elolf, Christian Strauss, Markus Otto, Alexander Mensch, Samir Abu-Rumeileh

**Affiliations:** 1 Department of Neurology, Martin-Luther-University Halle-Wittenberg, Halle (Saale), Germany; 2 Department of Neurosurgery, Martin-Luther-University Halle-Wittenberg, Halle (Saale), Germany; 3 Department of Radiology, Martin-Luther-University Halle-Wittenberg, Halle (Saale), Germany; 4 Department of Biotechnological and Applied Clinical Sciences, University of L’Aquila, L’Aquila, Italy; 5 Department of Stroke and Neuroscience, Charing Cross Hospital, Imperial College London NHS Healthcare Trust, London, UK; 6 Division of Brain Sciences, Department of Medicine, Hammersmith Campus, Imperial College London, London, UK

**Keywords:** Posterior reversible encephalopathy syndrome, PRES, Antibiotics, Adverse drug reactions, Metronidazole, Neurofilament light chain

## Abstract

Posterior reversible encephalopathy syndrome (PRES) is an acute neurological condition associated with different etiologies, including antibiotic therapy. To date, most data regarding antibiotic-related PRES are limited to case reports and small case series. Here, we report a novel case description and provide a systematic review of the clinico-radiological characteristics and prognosis of available cases of PRES associated with antibiotic therapy. We performed a systematic literature search in PubMed and Scopus from inception to 10 January 2024, following PRISMA guidelines and a predefined protocol. The database search yielded 12 subjects (including our case). We described the case of a 55-year-old female patient with PRES occurring one day after administration of metronidazole and showing elevated serum neurofilament light chain protein levels and favorable outcome. In our systematic review, antibiotic-associated PRES was more frequent in female patients (83.3%). Metronidazole and fluoroquinolones were the most reported antibiotics (33.3% each). Clinical and radiological features were comparable to those of PRES due to other causes. Regarding the prognosis, about one third of the cases were admitted to the intensive care unit, but almost all subjects (90.0%) had a complete or almost complete clinical and radiological recovery after prompt cessation of the causative drug. Antibiotic-associated PRES appears to share most of the characteristics of classic PRES. Given the overall good prognosis of the disease, it is important to promptly diagnose antibiotic-associated PRES and discontinue the causative drug.

**Supplementary Information:**

The online version contains supplementary material available at 10.1007/s10072-024-07545-1.

## Introduction

**Fig. 1:**
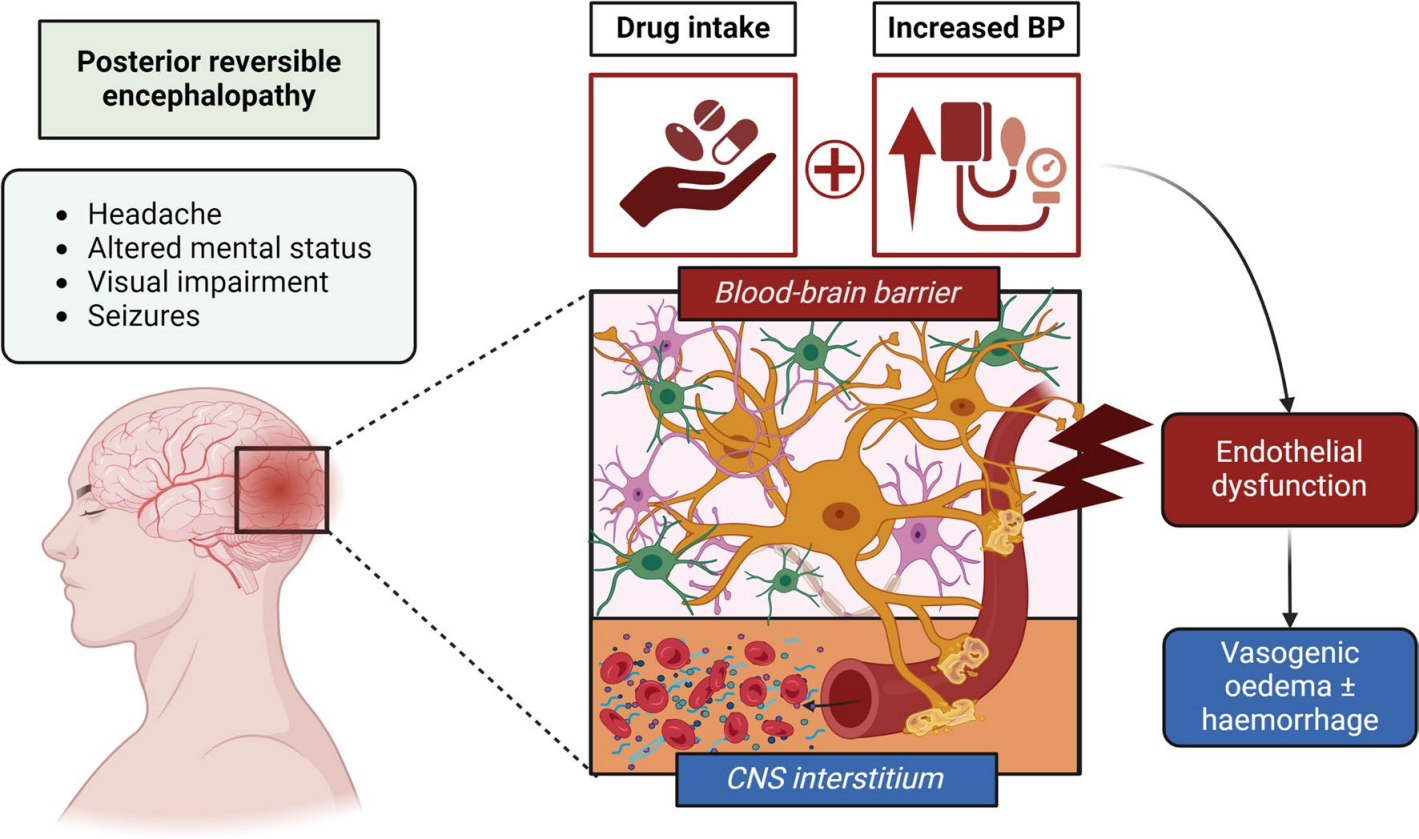
Clinical features and pathophysiological mechanisms of PRES associated with antibiotic and other drug administration. Disruption of the blood–brain barrier (BBB) with endothelial dysfunction (red box) leads to fluid leakage and vasogenic oedema with or without haemorrhage (blue box) in the central nervous system (CNS) interstitium. Drug intake (e.g., antineoplastic, immunomodulatory, and antimicrobial agents) might cause injury or inhibition of endothelial cell proliferation. Arterial hypertension is also a contributor in PRES pathophysiology. Involved risk factors are represented in red squares. Main clinical manifestations associated with PRES are summarized in the light blue box on the left part of the figure

Posterior reversible encephalopathy syndrome (PRES) is an acute neurological syndrome associated with subcortical vasogenic oedema, particularly in the posterior parieto-occipital regions. Typical clinical manifestations include non-specific symptoms such as headache and dizziness, but also more severe features such as altered consciousness, visual impairment and seizures [1, 2]. The pathophysiology of PRES is not fully understood yet but appears to be related to dysregulation of cerebral blood flow, cytokine release, disruption of the blood–brain barrier. This may result in endothelial damage and fluid leakage [2]. The posterior regions of the brain appear to be particularly susceptible to hyperperfusion and vasogenic oedema because of the relatively reduced sympathetic innervation in the posterior fossa, and consequently lower autoregulatory potential [1]. Indeed, in the majority of cases (approximately 75–80%) patients diagnosed with PRES showed moderate to severe increase of blood pressure (BP) values [3]. Other conditions that predispose to PRES include eclampsia/pre-eclampsia and renal failure, but also autoimmune or genetic disorders [1, 4–7]. In addition, the development of PRES may be secondary to the administration of drugs. A recent study based on the World Health Organization (WHO) global pharmacovigilance database identified 152 drugs significantly associated with PRES, most of which were antineoplastic, immunomodulatory, and antimicrobial agents [2]. These agents might cause injury or inhibition of endothelial cell proliferation [2] (Fig. 1).

To date, most data on antibiotic-associated PRES are limited to case reports and small case series. However, given the widespread use of antibiotics in daily clinical practice, it is important to better understand this rare but severe complication of antibiotics. This should also be emphasized in the scenario where non-specific symptoms related to PRES in antibiotic-treated patients may be overlooked and mistakenly attributed to underlying infections as well as other comorbidities that complicate hospitalisation. Furthermore, given the overall good prognosis of PRES with appropriate treatment, it is important to promptly identify potential predisposing factors and discontinue the agent in drug-associated PRES.

Considering all these issues, we aimed to describe a novel case of PRES after metronidazole administration and to perform a systematic review of the clinico-radiological characteristics and prognosis of available cases of PRES associated with antibiotic therapy.

## Case description

A 55-year-old woman with no history of immunological, renal or cardiovascular disease was admitted to our neurosurgery department for planned surgical removal of a right vestibular schwannoma causing long-standing right-sided hypoacusis. After the complete removal of the mass, a right-sided peripheral facial nerve palsy (Grade 4, according to the House-Brackmann score) [8] was noted as a postoperative complication. This was treated with eye protection and physiotherapy.

The patient received no antithrombotic or anticoagulant medication before and after surgery.

**Fig. 2:**
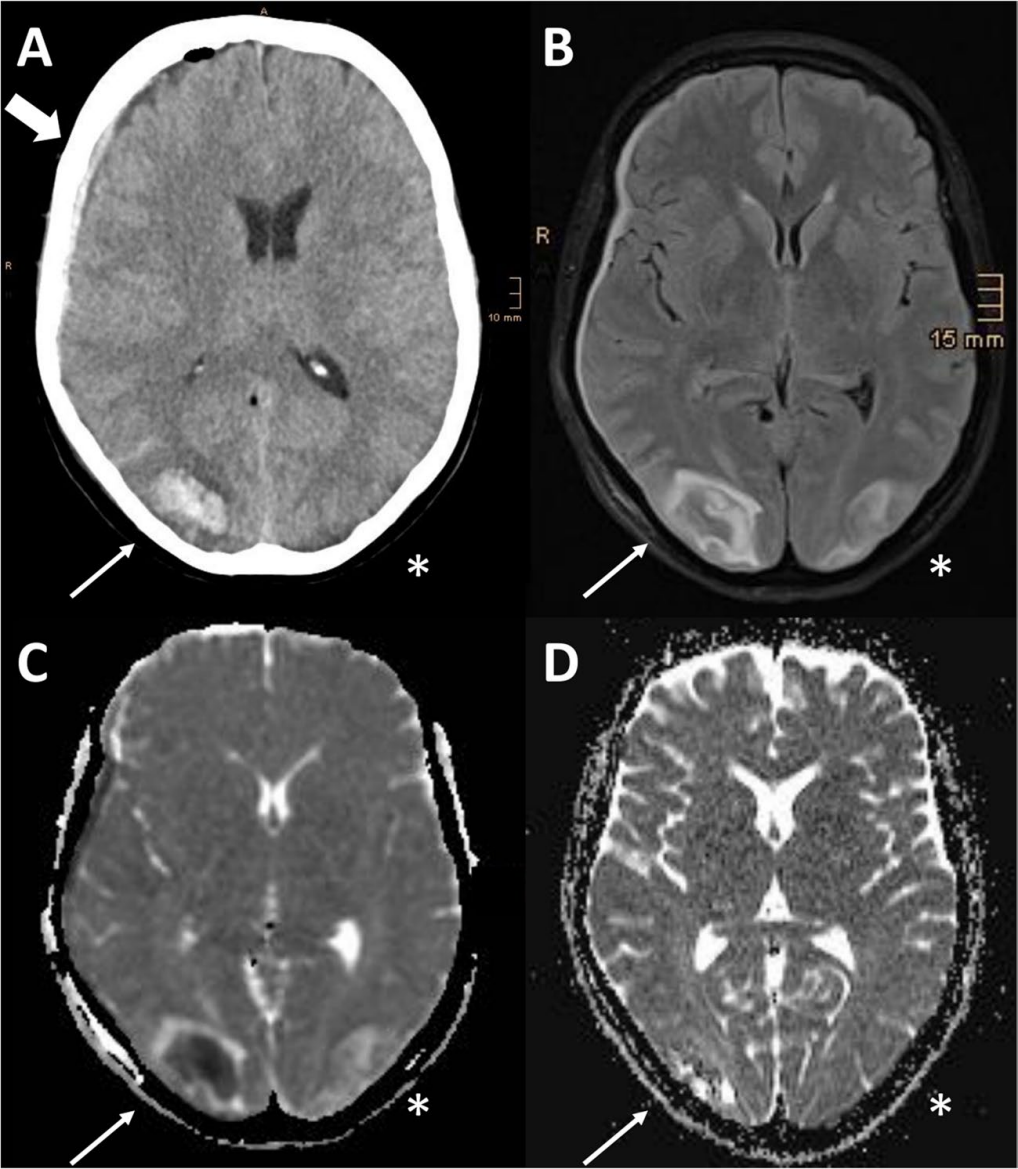
Imaging findings in our case of PRES after metronidazole administration. Native brain CT imaging showed A) right-sided occipital intracerebral haemorrhage with peripheral oedema (thin arrow), subdural bleeding (large arrow); left-sided grey-white matter junction oedema (*). MRI performed 2 days after clinical onset revealed bilateral occipital B) FLAIR (thin arrow and *) and C) ADC hyperintensities (thin arrow and *) indicating vasogenic edema. The relative right occipital hypointensity on the ADC map is related to the haemorrhage. D) Follow-up MRI after 2 months from clinical onset showed in the ADC map almost complete resolution of the vasogenic oedema and subtotal resorption of parenchymal haemorrhage (thin arrow and *). Abbrevations. ADC: apparent diffusion coefficient; CT: computed tomography; FLAIR: fluid-attenuated inversion recovery; MRI: magnetic resonance imaging; PRES: posterior reversible encephalopathy syndrome

Two days after successful surgery, the patient complained of abdominal pain and diarrhea. Microbiological testing revealed positive stool samples for *Clostridium difficile*, for which metronidazole (400 mg × 3/day) was administered. One day after starting metronidazole, the patient developed a diffuse mild headache (numerical rating scale 4/10), dizziness and nausea, followed by altered mental status with somnolence. Neurological examination revealed left-sided homonymous hemianopia without further focal deficits. BP was modestly elevated at clinical onset (158/60 mmHg). Computed tomography (CT) revealed a right occipital intracerebral haemorrhage with peripheral oedema, right subdural and subarachnoid bleeding (Fig. 2A) as well as contralateral grey-white matter junction oedema. Laboratory parameters indicated a mild inflammatory reaction (Supplementary Table 1). Renal function was unremarkable [glomerular filtration rate, GFR > 90 ml/min (normal range > 60), creatinine 62 micromol/L (normal range 44–809) and electrolytes [Na^+^ 144 mmol/l (normal range 136–145), K^+^ 4.0 mmol/l (normal range 3.4–4.5), Ca^++^ 2.19 mmol/l (normal range 2.15–2.50), Cl^−^ 107 (normal range 98–107)]. The patient was therefore admitted to the neurological intensive care unit (ICU) and the arterial hypertension was treated with continuous administration of urapidil and nimodipine. Over the next days, BP values remained normal without the need for intravenous antihypertensive medication. Headache was treated with metamizole and fentanyl transdermal patch. Magnetic resonance imaging (MRI), performed one day after symptom onset, showed subcortical T2, fluid-attenuated inversion recovery (FLAIR) and apparent diffusion coefficient (ADC) hyperintensities in occipital areas (Fig. 2B-C) without diffusion restriction signals reflecting vasogenic oedema (Supplementary Fig. 1). MRI including T2* gradient-echo sequences did not reveal findings associated with cerebral amyloid angiopathy [9]. Based on clinical and radiological findings, the diagnosis of PRES was made [1]. Given the previous association between PRES and metronidazole administration reported in the literature [10–12], metronidazole was discontinued 3 days after first administration and replaced by vancomycin (125 mg × 4/die). Neurofilament light chain protein (NfL) was measured in serum using the ELLA technology as previously described [13]. Serum NfL was massively elevated two days after symptom onset (166 pg/ml, normal values < 35 pg/ml according to our in-house reference values). Within 2 days of discontinuing metronidazole, the patient showed rapid clinical improvement with reduction of headache and improvement of mental status. At follow-up (4 days after symptom onset) serum NfL concentration were further increased (433 pg/ml).

After progressive improvement, the patient was discharged 13 days after the onset of PRES without hemianopia or other neurological sequelae, except for the unchanged right facial palsy. Follow-up MRI at approximately 2 months showed almost complete radiological recovery with resolution of the vasogenic oedema and subtotal resorption of parenchymal haemorrhage (Fig. 2D).

## Methods

We conducted a systematic study-level meta-analysis according to the Preferred Reporting Items for Systematic Reviews and Meta-Analyses (PRISMA) guidelines and a protocol agreed upon by all authors. Four authors (LB, SAR, LD, MF) searched for articles in publicly available literature databases (PubMed, Scopus) from inception to 10th January 2024. The search strategy was based on the combination of antibiotic names listed in the World Health Organization (WHO) Aware, Watch and Reserve (AWaRe) Classification 2021 [14] and "pres" or "posterior reversible encephalopathy syndrome" as either keywords or Medical Subject Heading (MeSH) terms (full search string is provided in the Supplementary materials). Reference lists and citing articles were also reviewed to increase the identification rate of relevant studies. Titles and abstracts were screened independently. Potentially relevant articles were acquired in full text and assessed for eligibility by the same four authors working in pairs. The final selection was shared among all the four authors. Disagreements were resolved by consensus.

Non-original records, studies with no full text available and articles in languages other than English were excluded. In addition, we included only studies that reported: 1) diagnosis of PRES [1, 2]; 2) recent administration of antibiotics and medical indication thereof; 3) information on symptoms and vital parameters at clinical onset; 4) at least one neuroimaging marker supporting the diagnosis of PRES [1, 2].

From the included studies, we extrapolated the following data, if available: 1) general information and clinical history of the patient, including all prescribed drugs and corresponding medical indications; 2) symptoms at clinical onset and after drug withdrawal; 3) vital and laboratory parameters; 4) therapeutic management of PRES; 5) neuroimaging findings at clinical onset and follow-up. The strength of the association between antibiotic prescription and PRES symptoms was assessed through the Naranjo Adverse Drug Reaction Probability Scale (NADRPS) [15].

## Results

By using the reported search string (Supplementary materials, Appendix), we identified a total of 232 articles published in literature. After duplicate removal, 187 articles were screened. Title and abstract screening led to the selection of 24 articles for full read, which brought us to a final number of 11 articles fulfilling the inclusion criteria and reporting one case each (Supplementary Table 1) [10–12, 16–23]. Full data on literature case reports (n = 11) and on our case are summarized in Table 1 and detailed in Supplementary Table 1.

**Table 1:** Demographic, clinical and radiological characteristics of PRES cases associated with antibiotic therapy (n = 12, including our case report).

General data	
World region (n)	Europe (4) East Asia (3) North America (3) South Asia (1) Middle East (1)
Age in years [mean ± sd (range)]	50.2 ± 22.3 (16–87)
Sex (n, %)	female (10, 83.3) / male (2, 16.7)
Prescribed antibiotic (n)	metronidazol (4) linezolid (2) rifampicin (2) ciprofloxacin (2) moxifloxacin (1) levofloxacin (1) daptomycin (1) teicoplanin (1) ceftriaxone (1) vancomycin (1)
Co-prescribed drugs (n)	immunosuppressive drugs (3) antihypertensive drugs (2) diuretic drugs (2) antiaggregation drugs (2) painkiller (1) other antibiotics (2) depressant drugs (1) none reported (5)
Indication for antibiotic prescription (n)	gastrointestinal infection (4) septic arthritis (3) pneumonia (2) infectious endocarditis (1) urinary tract infection (1) disseminated infection (1)
Pathogen reported (n)	*C. difficile* (1) *Enterococcus sp.* (1) *S. epidermidis* (1) *M. tubercolosis* (1) *A. actinomycetemconcomitans* (1) no pathogen reported (5)
Relevant comorbidities (n)	cardiovascular diseases (1 pulmonary embolism, 2 arterial hypertension, 1 deep venous thrombosis, 1 peripheral artery disease, 1 atrial fibrillation) lung diseases (1 chronic obstructive pulmonary disease) kidney diseases (1 acute kidney injury, 1 chronic kidney disease) metabolic diseases (2 diabetes mellitus, 1 hypothyroidism) rheumatological diseases (1 rheumatoid artritis) others (1 dyserythropoiesis, 1 anemia, 1 depression) none (5)
Clinical and radiological data at onset	
Time from drug intake to PRES onset (n)	within 1 h (1) within 1 week (7) within 2–3 weeks (4)
Neurological symptoms (n)	altered mental status (11) seizures (7) headache (6) visual disturbances (2 blurred vision, 1 bilateral visual loss) nausea (2) others (each reported once: vertigo, meningism, fatigue, myoclonus, dysarthria, dysphagia, tetanus)
Blood pressure at onset (n)	hypertension (6) normotension (5) hypotension (1)
Blood laboratory parameters (n)	high infection parameters (7) renal dysfunction (5) electrolyte disturbances (4)
Additional analyses (n)	electroencephalogram alterations (2 global slow activity, 1 no seizure-like activity, 1 bifrontal cortical dysfunction, 1 bilateral occipital sharp waves and slow waves bursts, 1 tempo-parietal theta focus) increased CSF proteins (2) cerebrospinal fluid pleocytosis (1)
Main radiological findings (n)	bilateral parieto-occipital T2/FLAIR hyperintensities (11) T2/FLAIR hyperintensities in other regions (1 cerebellum, 1 pons) other vascular lesions (1 bilateral infarcts in the basal ganglia)
Hospitalisation period	
Treatment approach of choice (n)	antihypertensive therapy (7) antiepileptic therapy (5) admission to ICU (4) need for mechanical ventilation (2) not reported (2)
Time interval to clinical improvement (n)	within 1 week (7) within 2 weeks (1) not reported (4)
Follow-up data	
Neurological sequelae (n)	full recovery (9) seizures (1) not reported (2)
Main neuroradiological findings (n)	complete radiological recovery (9) subtotal radiological recovery (1) not reported (2)
NADRPS (n)	1–4 possible (11) 5 probable (1)

### Demographic and clinical characteristics of reported cases

We performed the final analysis on 12 subjects, including our case. Patients had a mean age of 50.2 years (± standard deviation: 22.3 years; range: 16–87) and were predominantly female (10 of 12, 83.3%). Patients had multiple comorbidities, especially cardiovascular, renal and metabolic diseases (Table 1). The list of all prescribed antibiotics, their indications and other clinical information regarding the cases are reported in detail in Supplementary Table 1 and are summarized in Table 1. No pathogen-specific patterns were found, but gastrointestinal infections were most common (4 of 12, 33.3%). Of note, three articles [10, 11, 18] described the possible association of 2 antibiotics each with PRES. Metronidazole and fluoroquinolones were the most commonly reported antibiotics, each being administered in 4 cases (33.3%) (Table 1). The association between antibiotic administration and PRES, as assessed by the NADRPS score, was considered possible in 11 cases including our case report (NADRPS score 1–4, 91.7%) and probable in 1 case (NADRPS score 5, 8.3%). The types of infection and, when reported, the underlying pathogens were heterogeneous between studies and did not show any specific pattern (Table 1 and Supplementary Table 2).

### Clinical, laboratory and radiological findings at clinical onset

PRES manifestations started within one week of antibiotic administration in most cases (7 of 12 reports, 66.7%) and, most commonly, included altered mental status (11 of 12 reports, 91.7%), seizures (7 of 12 studies, 58.3%) and headache (6 of 12 reports, 50.0%), often in combination (Table 1). At presentation, patients often had elevated BP (> 140/90 mmHg) (6 of 12 reports, 50.0%), but PRES also occurred in the setting of normal (90–140/60–90 mmHg) (5 of 12 reports, 41.7%) or decreased BP (< 90/60 mmHg) (1 of 12 reports, 8.3%). Laboratory changes related to the ongoing inflammatory process were found in 7 of 12 reports (58.3%), while electrolyte disturbances and renal dysfunction were described in 4 (33.3%) and 5 of 12 reports (41.7%), respectively. In addition, pathological electroencephalogram (EEG) findings were  reported in 4 out of 12 subjects (33.3%). In all cases, MRI performed during the acute phase showed posterior hyperintensities in occipital areas (11 of 12 reports, 91.7%) or the pons (1 of 12, 8.3%), compatible with PRES. Our case was the only one to show lobar haemorrhage occurring together with bilateral vasogenic oedema. ICU admission and mechanical ventilation were required in 4 of 12 (33.3%) and 2 of 12 (16.7%) cases, respectively. Antiepileptic therapy was administered in 5 of 12 patients (41.7%) (Table 1).

### Follow-up data

The vast majority of subjects showed significant clinical improvement within a few days after antibiotic cessation, with no neurological sequelae at follow-up (9 out of 10 cases reported, 90.0%, in 2 articles no follow-up data were reported). Furthermore, follow-up imaging showed complete radiological recovery in almost all cases (9 out of 10 reported cases, 90.0%, in 2 articles no follow-up data were reported) (Table 1).

## Discussion

In this study, we described a case of PRES associated with recent antibiotic therapy and reviewed similar reports in the literature. Our main findings suggest that PRES associated with antibiotic therapy: 1) can occur at any age and may affect both sexes, with a higher frequency in female patients; 2) is often, but not necessarily, associated with arterial hypertension; 3) occurs within a few days of drug administration, presents with altered mental status and/or seizures, and shows pathological MRI findings in the parieto-occipital areas, as well as heterogeneous EEG changes; 4) may require admission to ICU and mechanical ventilation in about one third of cases, but in the vast majority of patients show complete or almost complete clinical and radiological recovery after prompt discontinuation of the drug; 5) may not be antibiotic or pathogen specific, although metronidazole and fluoroquinolones were the most commonly reported antibiotics.

Overall, our findings are in line with previous literature on the pathophysiological and clinical features of PRES; indeed, previous studies reported also on the female predominance in drug-associated PRES cases [2]. Further, the elevation of arterial BP is thought to be the driving pathological mechanism in most cases of PRES, leading to cerebral hyper-perfusion [1]. In our study, the frequencies of patients with hyper-, normo- and hypotension well reflects those of previous reports (i.e., approximately 30–50% of PRES cases without elevated BP values) [3]. In addition, altered mental status (92%), headache (50%) and seizures (58%) were common findings in the patient group analyzed, largely reflecting the prevalences reported in other PRES cohorts [1, 5, 24–26]. To date, there are no data to support specific antiepileptic treatment regimens for PRES compared with those used in other conditions. Furthermore, although seizures in PRES may be associated with a worse clinical outcome [26] in most cases they do not recur [27]. Prospective studies are therefore urgently needed to assess the impact of antiepileptic therapy on outcomes in PRES.

Brain MRI is currently the gold standard for the diagnosis of PRES [1]. Although parieto-occipital hyperintensities are the classic neuroradiological features of PRES [1], concomitant intraparenchymal or subarachnoid haemorrhages, like in our case report, have been described in literature in patients with PRES not associated with antibiotics [24, 28–31]. Our reported case is therefore peculiar because it is the first to be described. As previously hypothesised, reperfusion injury due to vasoconstriction or pial rupture secondary to hypertension appears to be the leading pathogenic mechanism in PRES-associated intracranial haemorrhage [24].

NfL is a marker of neuroaxonal damage in primary and non-primary neurological diseases [32]. To date, only one study has investigated and demonstrated an increase in serum NfL in patients with PRES [33]. Our case report supports these observations, but we cannot exclude the significant confounding effects of cerebral haemorrhage and neurosurgical treatment on NfL concentrations as described previously [13, 34].

Regarding the prognosis, most cases of PRES associated with antibiotic therapy or other drugs showed a complete or almost complete clinical recovery [35], however also permanent disability and death have been reported in few cases [36].

Of note, metronidazole and fluoroquinolones turned out to be the antibiotics more frequently associated with PRES in our systematic review. Some authors have hypothesized that metronidazole treatment may lead to autonomic dysfunction and, thus, to PRES. Indeed, a case of metronidazole-induced sympathetic autonomic dysfunction has been described [37]. Interestingly, metronidazole therapy has previously been related also to another neurological syndrome, namely metronidazole-induced encephalopathy (MIE). Indeed, MIE can present with encephalopathic symptoms as well as seizures mimicking PRES [38]. However, unlike PRES, MIE often occurs weeks/months after drug ingestion, presenting primarily with motor symptoms (e.g., dysarthria, postural instability, and oculomotor dysfunction) due to the involvement of cerebellar and brainstem structures [38]. In addition, in MIE, MRI shows characteristic reversible symmetrical T2/FLAIR hyperintense lesions of the dentate nuclei in the vast majority of patients [38]. Taking in to account these findings, we suggest that a misdiagnosis might possibly explain the low number of PRES cases associated with metronidazole prescription in studies of pharmacovigilance databases [38].

With regard to fluoroquinolones, these drugs are known to cause central and peripheral neurotoxicity with variousmanifestations such as headache and seizures [39], but the underlying mechanisms of fluoroquinolone-associated PRES remain unclear [12]. Nevertheless, given the frequent use of these antibiotics and the relatively modest absolute risk of adverse neurological effects following fluoroquinolone administration [39], it is important to consider PRES early in the differential diagnosis of acute neurological symptoms emerging on this therapy. Furthermore, in clinical practice it can sometimes be difficult to understand whether the antiinfective therapy or a specific infectious disease itself is the trigger of PRES. For example, in a literature case of PRES in a human immunodeficiency virus (HIV)-infected patient on antiretroviral therapy, both the pathogen and the drug were discussed to play a pathogenic role [40].

The main limitation of our review is its retrospective nature; therefore, our findings should be confirmed by pharmacovigilance investigations, or by prospective or translational studies to identify the pathophysiological mechanisms underlying antibiotic-associated PRES. Moreover, further studies on PRES associated with other classes of drugs, especially antiviral and antineoplastic agents, are needed.

In conclusion, the occurrence of acute or subacute neurological symptoms after antibiotic treatment should raise the clinical suspicion towards PRES. MIE should be considered in the differential diagnosis in case of metronidazole prescription [41]. Brain MRI allows rapid and accurate identification of vasogenic oedema consistent with PRES, which could otherwise be missed on CT alone. Indeed, in our case description, the peculiar presence of concomitant intracerebral haemorrhage could have been misleading without advanced imaging. Early recognition and appropriate management of PRES is necessary to eliminate any predisposing causes. This would lead to a shorter recovery time, reduced need for intensive care and overall better clinical outcomes.

### Supplementary Information

Below is the link to the electronic supplementary material.

Supplementary file1 (DOCX 1062 KB)

## Data Availability

The datasets generated during and/or analysed during the current study are available from the corresponding author on reasonable request.
