# Supplementary material for: Posterior reversible encephalopathy syndrome associated with antibiotic therapy: a case report and systematic review
Source: Neurol Sci. 2024 Apr 29;45(9):4151–9. doi: 10.1007/s10072-024-07545-1 (PMC11306538; doi:10.1007/s10072-024-07545-1)
Supplement: Supplementary file 1 — Supplementary file1 (DOCX 1062 KB) [file 10072_2024_7545_MOESM1_ESM.docx]

**SUPPLEMENTARY MATERIALS**

**Appendix.** **Search string.**

| (pres OR posterior reversible encephalopathy syndrome) AND (antibiotic OR antibiotics OR amikacin OR amoxicillin OR clavulanic acid OR ampicillin OR arbekacin OR aspoxicillin OR azidocillin OR azithromycin OR azlocillin OR aztreonam OR bacampicillin OR bekanamycin OR benzylpenicillin OR biapenem OR brodimoprim OR carbenicillin OR carindacillin OR carumonam OR cefacetrile OR cefaclor OR cefadroxil OR cefalexin OR cefaloridine OR cefalotin OR cefamandole OR cefapirin OR cefatrizine OR cefazedone OR cefazolin OR cefbuperazone OR cefepime OR cefixime OR cefmenoxime OR cefmetazole OR cefodizime OR cefoperazone OR cefotaxime OR cefoxitin OR ceftazidime OR ceftezole OR ceftriaxone OR cefuroxime OR chloramphenicol OR chlortetracycline OR cinoxacin OR ciprofloxacin OR clarithromycin OR clindamycin OR cloxacillin OR colistin OR daptomycin OR delafloxacin OR dibekacin OR dicloxacillin OR doxycycline OR ertapenem OR erythromycin OR flucloxacillin OR fosfomycin OR furazidin OR fusidic acid OR gemifloxacin OR gentamicin OR grepafloxacin OR hetacillin OR imipenem OR isepamicin OR josamycin OR kanamycin OR lascufloxacin OR latamoxef OR lefamulin OR levofloxacin OR lincomycin OR linezolid OR lomefloxacin OR meropenem OR metacycline OR metampicillin OR meticillin OR metronidazole OR mezlocillin OR micronomicin OR midecamycin OR minocycline OR moxifloxacin OR miocamycin OR nafcillin OR nemonoxacin OR neomycin OR netilmicin OR norfloxacin OR ofloxacin OR oleandomycin OR omadacycline OR ornidazole OR oxacillin OR oxytetracycline OR panipenem OR pazufloxacin OR pefloxacin OR penamecillin OR piperacillin OR pivampicillin OR plazomicin OR polymyxin b OR propicillin OR ribostamycin OR rifabutin OR rifampicin OR rifamycin OR rifaximin OR rokitamycin OR rolitetracycline OR rosoxacin OR secnidazole OR sisomicin OR sitafloxacin OR sparfloxacin OR spectinomycin OR spiramycin OR streptomycin OR sulbactam OR sulfadiazine OR sulfadiazine OR sulfamethoxazole OR tazobactam OR tebipenem OR teicoplanin OR temafloxacin OR trimethoprim OR tetracycline OR tigecycline OR tinidazole OR tobramycin OR troleandomycin OR ticarcillin OR trovafloxacin OR vancomycin) |
| --- |

**Supplementary Figure 1. PRISMA flow chart for study selection.**


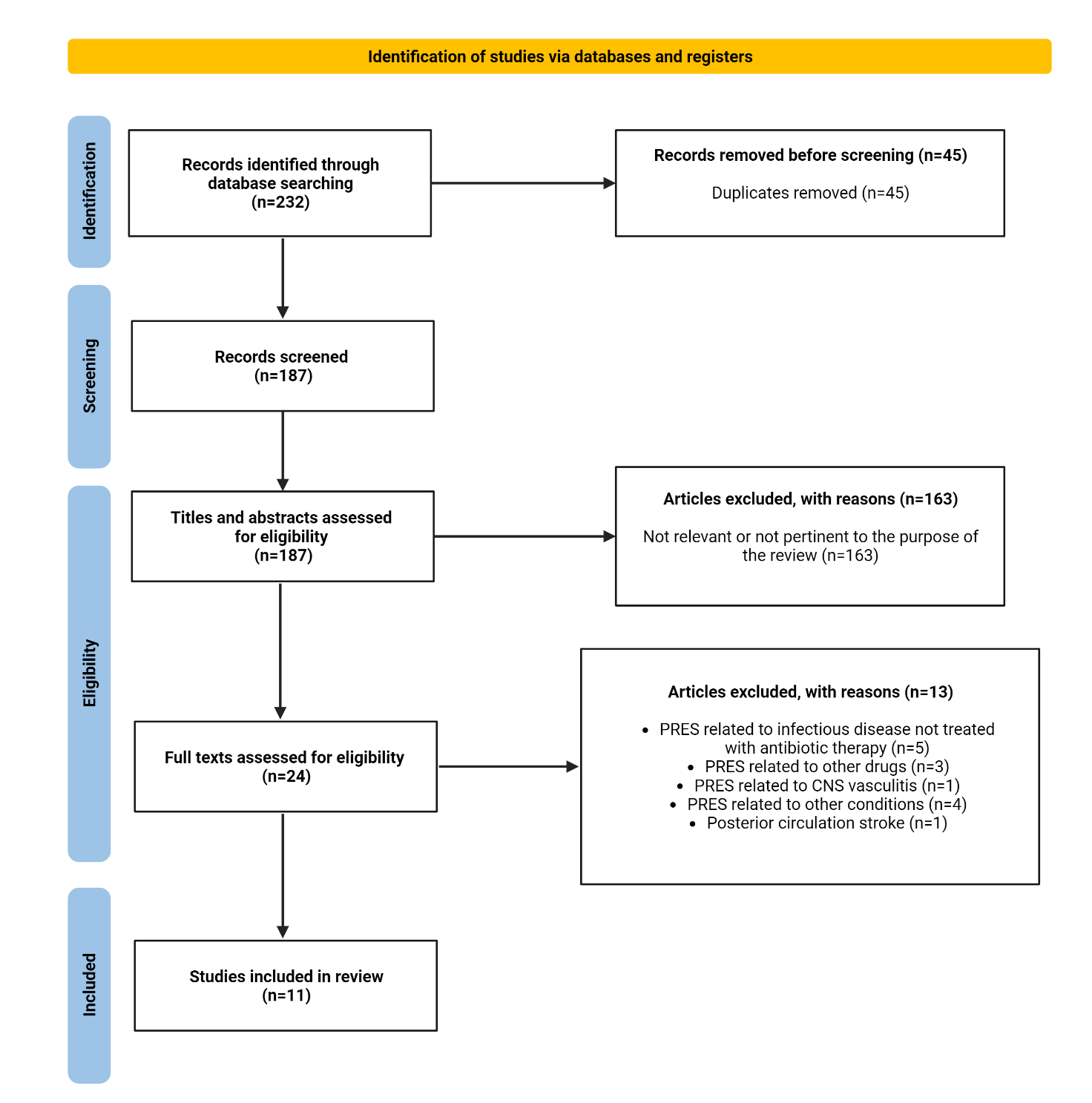


**Supplementary Figure S1.** Diffusion weighted imaging (DWI) sequences during the acute phase (A) and at follow-up (B).

**
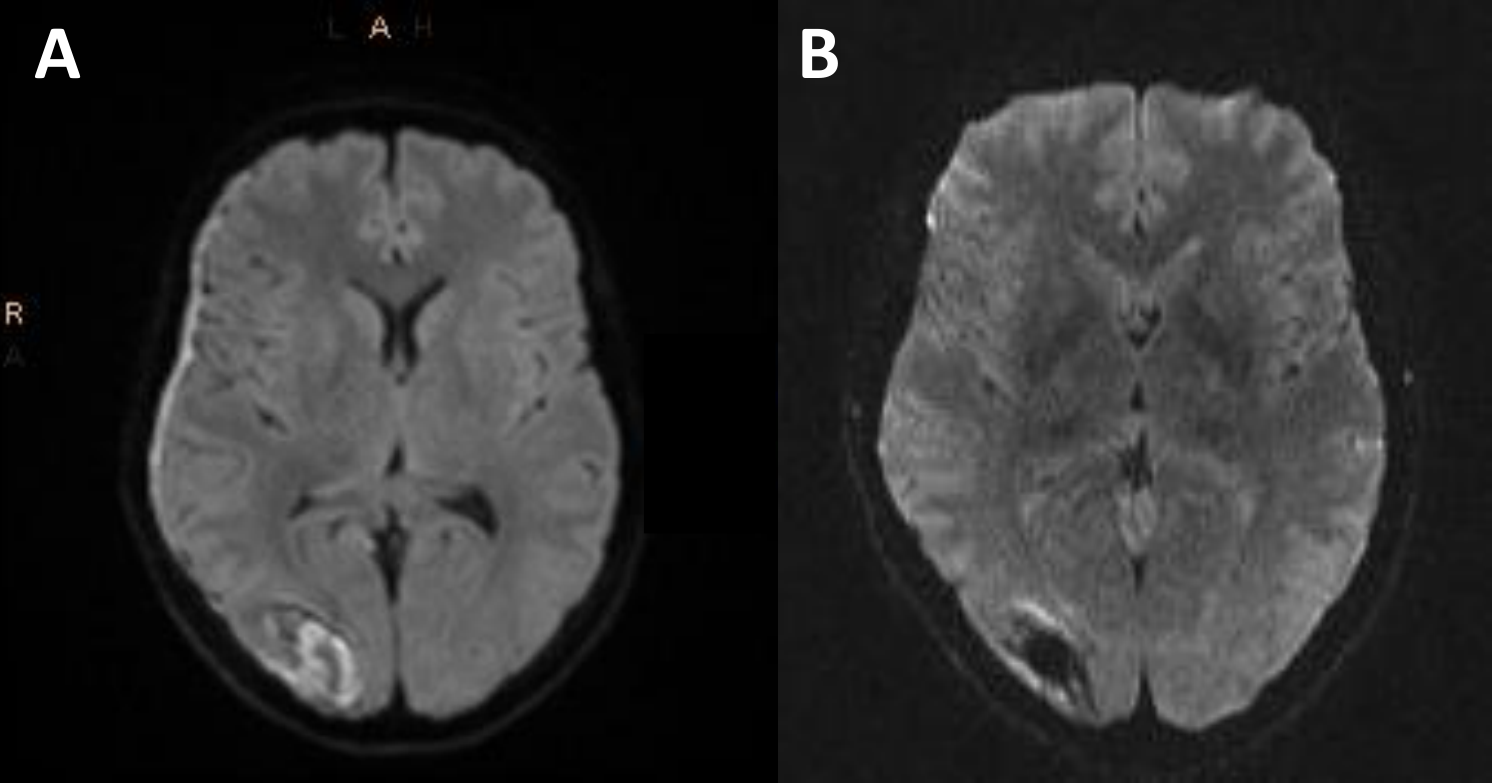
**

**Supplementary Table 1.** Studies reporting on PRES cases associated with antibiotic therapy.

| **Reference** | **Country** | **Age** | **Sex** | **Prescribed antibiotics and therapy duration** | **Co-prescribed drugs** | **Indication for drug prescription** | **Relevant comorbidities** | **Neurological symptoms at onset** | **Time interval from initial drug intake and first PRES-associated symptoms** | **Neurological sequelae at follow-up** | **Time interval after drug withdrawn and first clinical improvement** |
| --- | --- | --- | --- | --- | --- | --- | --- | --- | --- | --- | --- |
| Barba et al. 2024 | Germany | 55 | female | Metronidazole (400 mg x 3/die) | Dexamethason (8 mg x 1/die)  Pantozol (40 mg x 1/die)  Metamizole ( 750 mg x 3/die)  L-thyroxin (75 μg x 1/die) | diarrhea due to *C. difficile* infection | none | altered mental status, vertigo, nausea, headache | 1 day | none | 2 days |
| Tomar et al. 2022 Tropical Doctor | India | 45 | female | Linezolid (600 mg x 1/die) | Dexamethasone (2 g x 3/die)  Moxifloxacin (400 mg x 1/die)  Terizidone (250 mg x 3/die)  Ethambutol (1 g x 1/die)  Amikacin (1g x 1/die) | Disseminated tubercolosis | not reported | altered mental status, intact motor function | 2 days | none | 2 days |
| Ali et al. 2013 BMJ Case Reports | Saudi Arabia | 16 | male | Ciprofloxacin (no dose reported) | Diazepam (10mg x 1/die)  Haloperidol (no dose reported)  Midazolam (no dose reported) | Pneumonia (no pathogen reported) | not reported | headache,  altered mental status, tonic-clonic seizures, neck stiffness | 3 days | none | 3 days |
| Bitar De Zayas-Enriquez et al. 2019 Case reports in neurological medicine | United Kingdom | 60 | female | Daptomycin (4mg/kg x 1/die) | Prednisolon (25mg x 1/die) (discontinued the day before)  Others (no posology reported): aspirin, simvastatin, metformin, leflunomide, hydroxychloroquine, epoetin, folic acid, solifenacin, chlorphenamine, omeprazole, ranitidine, quinine sulphate, cocodamol, pregabalin, tramadol, citalopram | Urinary tract infection from glycopeptide-resistent *Enterococcus sp.* | stage III acute kidney injury,  rheumatoid arthritis, COPD, pulmonary embolism, diabetes mellitus, dyserythropoiesis, depression | tonic-clonic seizures for 2 minutes three times within 2h | 50 minutes | partial seizures after the first episodes (unspecific time period) | not reported |
| Cordeanu et al. 2017 Fundamental & Clinical Pharmacology | France | 67 | female | Rifampicin (600mg x 2/die)  Teicoplanin (400mg x 1/die) | Nicardipine (50mg x 2/die)  Irbesartan (300mg x 1/die)  Hydrochlorotiazide (25mg x 1/die)  Urapidil (60mg x 2/die)  Metformin (500mg x 2/die)  Atorvastatin (20mg x 1/die)  Aspirin (75mg x 1/die)  Esomeprazole (20mg x 1/die)  Alprazolam (0.25mg x 1/die) | Septic arthritis of the knee (no pathogen reported) | arterial hypertension, chronic renal impairment, deep venous thrombosis, peripheral artery disease, diabetes mellitus | status epilepticus (no symptoms specified) | 6 days | not reported | not reported |
| Gilbert et al. 2019 Hospital Pharmacy | USA | 75 | female | Ciprofloxacin (500mg x 2/die)  Metronidazole (500mg x 2/die) | none reported | Abdominal abscess with diverticulitis (no pathogen reported) | arterial hypertension, anemia, hypothyroidism | altered mental status, fatigue, nausea and vomiting, seizure-like activity | 3 weeks | none | 1-4 days |
| Hosaka et al. 2023 Neurological sciences | Japan | 87 | female | Metronidazole (500mg x 3/die)  Levofloxacin (250mg x 1/die) | none reported | Liver abscesses (no pathogen reported) | none reported | altered mental status, paresis and myoclonus of the right upper limb, fever, dysarthria, dysphagia | 2 weeks | not specified | not reported |
| Kikuchi et al. 2016 Internal Medicine | Japan | 25 | female | Metronidazole (no dose reported) | Cefazolin (2g/die)  Vancomycin (2g/die)  5-ASA (2.25g/die)  Prednisolone (60mg/die) | Ulcerative colitis | none reported | altered mental status, tetanus | 1 day | none | 1 day |
| Nagel et al. 2007 Arch Neurol | Germany | 71 | female | Linezolid (600mg x 2/die)  Rifampicin (600mg x 1/die) | Omeprazole (20mg/die)  Ramipril (10mg/die)  Hydrochlorotiazide (25mg/die)  Torasemide (10mg/die)  Metoprolol (90mg/die)  Iodine (200mg/die)  Nadroparin (5700IU/die)  Metamizole (1000mg/die)  Allopurinol (300mg/die) | Infected hip prothesis due to multiresistent *S. epidermidis* | arterial hypertension, atrial fibrillation | altered mental status, holocephalic headache, blurred vision, focal motor seizure of the right upper limb (focal motor status epilepticus with Todd paresis after 6 days from clinical onset) | 5 days | none | few days |
| Kim et al. 2021 J Clin Neurol | USA | 32 | female | Vancomycin (no dose reported) | none reported | cellulitis of the right hip (no pathogen reported) | none | bilateral visual loss, altered mental status, seizures | 12 days | none | within 2 weeks |
| Nasu et al. 2017 Nephrology | Japan | 37 | female | Ceftriaxone (no dose reported) | none reported | infectious endocarditis (*Aggregatibacter actinomycetem-concomitans*) | chronic glomerulonephritis, anemia | headache, nausea, altered mental status | 14 days | none | not reported |
| Reyes et al. 2017 BMJ Case Rep | USA | 32 | man | Moxifloxacin (no dose reported) | none reported | community-acquired pneumonia (no pathogen reported) | acute kidney injury, nephritic syndrome, post-streptococcal glomerulonephritis | headache, blurred vision, altered mental status | 1 day | none | 7 days |

Table 1 (continuation).

| **Reference** | **Vital parameters at clinical onset** | **Blood laboratory parameters at clinical onset** | **Relevant additional analyses** | **Treatment approach of choice** | **Neuroimaging features at clinical onset** | **Neuroimaging features after drug discontinuation** | **NADRPS*** |
| --- | --- | --- | --- | --- | --- | --- | --- |
| Barba et al. 2024 | BP: 158/90  HF: 76/ min | CRP: 14.7 mg/l  D-dimer: 0.78 μg/ml | none | ICU admission, anthypertensive therapy (nimodipine and urapidil), metronidazol discontinuation and substitution with vancomycin | CT: right-sided occipital intracerebral haemorrhage and subarachnoidal haemorrhage and left-sided subcortical oedema.  MRI: bilateral T2/FLAIR hyperintensities in parieto-occipital lobes. | MRI after 2 months: subcomplete recovery | 4 - possible |
| Tomar et al. 2022 Tropical Doctor | BP: 134/84 mmHg | Mg^++^: 0.97 mEq/L  no alterations in hemotological, liver or renal function tests | - | not reported | MRI: symmetrical bilateral parieto-occipital T2/FLAIR hyperintensities, bilateral vasculitis infarcts in the basal ganglia | MRI after 2 weeks: complete recovery | 3 – possible for linezolid  2 – possible for moxiflocacin and terizidine |
| Ali et al. 2013 BMJ Case Reports | BP: 110/55 mmHg  HF: 110 bpm | K^+^: 2.7 mEq/L  pH: 7.475  WBC: 13000/μl  CK: 402 IU/L | Normal CSF analysis  Normal findings at ocular fundus examination  No seizure-like activity at EEG | ICU admission, antihypertensive (perindopril) and antiepileptic (diazepam) therapy | MRI: T2-hyperintensities in occipital areas bilaterally and in the left cerebellum | MRI after 2 weeks: complete recovery | 3 – possible |
| Bitar De Zayas-Enriquez et al. 2019 Case reports in neurological medicine | mean BP: 130 mmHg  SaO2: 94-97% | Hb: 10.5 g/dl  urea: 13.8 mmol/L  creatinine: 228 μmol/L  eGFR: 19 ml/min  CRP: 14 mg/dl | EEG: frequent brief runs of generalized semirhythmical slow activity with sharp components | ICU admission, intubation and mechanical ventilation, antihypertensive therapy (labetalol) | CT: hypodensities in both occipital lobes and in the right frontal lobe  MRI: bilateral FLAIR hyperintensities in occipital lobes | MRI after 2 weeks: almost complete recovery | 2 – possible |
| Cordeanu et al. 2017 Fundamental & Clinical Pharmacology | BP: 186/85 mmHg | urine proteins: 5.8 g/24h | - | Nicardipine discontinuation and replacement with alfuzosin, no specific anti-seizures therapy reported | MRI: diffuse cortical and subcortical hyperintensities bilaterally in the parieto-occipital lobes at FLAIR imaging | no follow-up | 2 – possible |
| Gilbert et al. 2019 Hospital Pharmacy | BP: hypotension | creatinine: 1.8 mg/dl | EEG: diffuse encephalopathic pattern with bifrontal cortical dysfunction | ICU admission, intubation and mechanical ventilation, antiepileptic therapy (levetiracetam), ciprofloxacin discontinuation and replacement with aztreonam and vancomycin | MRI: bilateral T2/FLAIR hyperintensities in occipital lobes | MRI after 18 days: complete recovery | 5 – probable for both ciprofloxacin and metronidazole  (reported) |
| Hosaka et al. 2023 Neurological sciences | normal BP, HF and SaO2 | ↑ CRP  ↓ Na^+^  normal renal and liver function | negative blood cultures  CSF: pleocytosis and increased protein concentration  EEG: bilateral occipital sharp waves and slow waves bursts | not reported | MRI: bilateral FLAIR hyperintensities in the occipital lobes | MRI after 6 weeks: almost complete recovery | 4 – possible for metronidazole  3 – possible for levofloxacin |
| Kikuchi et al. 2016 Internal Medicine | BP: 140/90 mmHg (100/60 mmHg the day before) | WBC: 14300/ μl  Hb: 10 g/dl  BUN: 27.5 mg/dl  Creatinine: 0.99 mg/dl  CRP: 0.99 mg/dl | normal CSF analysis  EEG: global slow activities | Aministration of phenytoin to prevent tetanus recurrence, antiedema therapy (glycerol) | MRI: bilateral FLAIR hyperintensities in the occipital and parietal lobes | MRI after 8 days: complete recovery | 3 – possible |
| Nagel et al. 2007 Arch Neurol | BP: 155/95 mmHg  HF: 102 bpm | CRP: 99 mg/dl | CSF: normal cell count (1 cell/ul), increased protein level (1.06 g/L), increased CSF/serum albumin ratio (16, reference range <9), no oligoclonal bands  EEG: right temporo-parietal theta focus | Antihypertensive (metoprolol, dihydralazine and urapidil) and antiepileptic (phenytoin) therapy, linezolid discontinuiation and replacement with fosfomycin | MRI: bilateral FLAIR hyperintensities in occipital lobes | MRI after 14 days: complete recovery | 2 – possible for linezolid  1 – possible for rifampicin |
| Kim et al. 2021 J Clin Neurol | BP 107-156 / 68 – 108 mmHg | creatinine: 3.22 mg/dl | - | Antyhypertensive and antiepileptic (levetiracetam and phenytoin) therapy | MRI: bilateral occipital and posterior parietal T2/FLAIR hyperintensities | MRI after 7 days: complete resolution | 3 – possible |
| Nasu et al. 2017 Nephrology | BP 160/80 mmHg | D-dimer: 8.8 μg/ml | - | Antyhypertensive therapy | MRI: T2 hyperintensities in the pons | MRI after 14 days: residual T2 hyperintensities in the pons | 3 – possible |
| Reyes et al. 2017 BMJ Case Rep | BP 138/98 mmHg | WBC count: 13320/μl  K^+^: 5.2 mmol/l  creatinine: 2 mg/dl | - | Antyhypertensive (amlodipin) and antiepileptic (phenytoin) therapy | CT: decreased bilateral white matter attenuation in parieto-occipital and frontoparietal areas  MRI: bilateral FLAIR hyperintensities in parietal and occipital lobes | none | 3 – possible |

**List of abbreviations.** 5-ASA: 5-aminosalicylate acid; BP: blood pressure; BUN: blood urea nitrogen; CK: creatinine kinase; COPD: chronic-obstructive pulmonary disease; CRP: C-reactive protein; CSF: cerebrospinal fluid; CT: computed tomography; DWI: diffusion-weighted imaging; EEG: electroencephalogram; eGFR: estimated glomerular filtration rate; EVD: external ventricular rain FLAIR: fluid-attenuated imaging recovery; Hb: hemoglobin; HF: heart frequency; ICU: intensive care unit; K^+^: potassium; Mg^++^: magnesium; MRI: magnetic resonance imaging; Na^+^: natrium; NADRPS: Naranjo adverse drug reaction probability scale; SaO2: oxygen saturation: WBC: white blood cells.

*The NADRPS scores reported in the table were estimated according to the information provided in the study except when already declared (“reported” in the table).
